# Acute ataxia in children: etiological spectrum and clinical characteristics

**DOI:** 10.3389/fped.2025.1613558

**Published:** 2025-11-13

**Authors:** Qing Zhao, Chao Gao, Lihui Wang, Chong Liu, Suzhen Sun, Baoguang Li

**Affiliations:** 1 Department of Neurology, Hebei Children’s Hospital, Shijiazhuang, Hebei, China; 2 Department of Neurology, The First Hospital of Hebei Medical University, Shijiazhuang, Hebei, China

**Keywords:** acute ataxia, children, etiology, autoantibodies, clinical characteristics

## Abstract

**Background:**

Acute ataxia is one of the most common movement disorders in children, characterized by complex etiologies, some of which may lead to disability or life-threatening complications. Early diagnosis and intervention are therefore crucial.

**Objective:**

This study aimed to investigate the etiological spectrum and clinical characteristics of children presenting with acute ataxia as the initial symptom.

**Methods:**

A retrospective analysis was conducted on children hospitalized at Hebei Children's Hospital between January 2018 and December 2024, all of whom exhibited acute ataxia as the primary manifestation. Clinical data, including etiology, age distribution, and laboratory findings, were systematically reviewed and analyzed.

**Results:**

A total of 257 children were included, with a male-to-female ratio of 1.14:1 and a median age of onset of 3 years (range: 10 months to 14 years). Initial screening of 315 records identified 58 patients for exclusion. Etiologies varied by age: infants/toddlers (0–3 years) showed acute postinfectious cerebellar ataxia (APCA, 66%), drug intoxication (14%), and acute disseminated encephalomyelitis (ADEM, 8%); preschoolers (4–6 years) had APCA (63%), ADEM (14%), and acute cerebellitis (AC, 8%); school-aged children (7–14 years) presented APCA (48%), AC (21%), and drug intoxication (14%). AC patients exhibited later onset, longer duration from symptom onset to hospital presentation, and more frequent neurological symptoms (encephalopathy, headache, vomiting) compared to APCA. Cerebrospinal fluid (CSF) nucleated cell counts were elevated in AC. Among APCA cases, CSF oligoclonal band (OCB)-positive patients had later onset, extended hospitalization, higher CSF nucleated cell counts, and higher relapse rates. Autoantibody screening in a subset of 135 patients identified CSF OCB, serum myelin basic protein (MBP), myelin oligodendrocyte glycoprotein (MOG) immunoglobulin G (IgG), GQ1b, and aquaporin-4 (AQP4) antibodies as common markers.

**Conclusion:**

The etiology of acute ataxia in children varies significantly by age, necessitating tailored diagnostic approaches. For cases suggestive of central nervous system demyelination or recurrent ataxia, comprehensive evaluations, including autoantibody testing, tumor screening, and genetic/metabolic assessments, should be considered to guide management and improve outcomes. CSF OCB positivity may identify an APCA subgroup with more pronounced inflammatory features and a potentially higher relapse risk.

## What is known

- Acute ataxia in children presents with a diverse etiological spectrum, where acute postinfectious cerebellar ataxia (APCA) is frequently reported.
- The predominant causes of acute ataxia in children often vary with age.
- Immune-mediated ataxias, including those associated with myelin oligodendrocyte glycoprotein (MOG) antibodies, can manifest with varied clinical presentations and may have a relapsing course.

## What is new

- This study provides a large single-center cohort analysis of acute ataxia in Chinese children, detailing age-specific etiological patterns and identifying enteroviruses as prominent post-infectious pathogens, reflecting regional infectious disease landscapes and the impact of local vaccination programs.
- Cerebrospinal fluid (CSF) oligoclonal band (OCB) positivity in APCA is associated with an older age of onset, prolonged hospitalization, higher CSF cell counts, and a significantly increased risk of relapse, suggesting a subgroup with more pronounced neuroinflammation.
- MOG antibodies are relevant not only in acute disseminated encephalomyelitis (ADEM) but also in a subset of children presenting with isolated cerebellar ataxia, some of whom experience a relapsing course, even with normal initial MRI findings.
- A detailed comparison between APCA and acute cerebellitis (AC) highlights distinct clinical and laboratory features aiding differential diagnosis, with AC patients demonstrating older onset age, more frequent and severe neurological symptoms, and higher CSF pleocytosis.

## Introduction

Acute ataxia is a frequent neurological manifestation in children, characterized by sudden-onset incoordination of gait, posture, or limb movements. Its etiological spectrum is highly heterogeneous, encompassing immune-mediated disorders (e.g., APCA, ADEM, antibody-associated encephalitis), infections (e.g., viral cerebellitis), intoxications (e.g., antiepileptics, alcohol), structural lesions (e.g., posterior fossa tumors, stroke), metabolic derangements (e.g., maple syrup urine disease), and genetic conditions (e.g., episodic ataxias) (1). While many cases are self-limiting, certain etiologies—such as brainstem tumors or autoimmune encephalitis—may lead to severe neurological sequelae or even mortality if untreated (1, 2). Thus, prompt etiological differentiation is paramount for tailored management.

The diagnostic challenge lies in the overlapping clinical presentations across diverse causes (2). For instance, APCA, the most common etiology, typically follows a viral prodrome and is characterized by pure cerebellar dysfunction, whereas ADEM or AC often presents with additional neuroinflammatory features (e.g., altered consciousness, seizures) (3). Recent advances in neuroimmunology have identified autoantibodies (e.g., MOG IgG, GQ1b) as key biomarkers for immune-mediated ataxias, yet their prevalence and clinical correlates in pediatric cohorts remain underexplored (4, 5). Similarly, the prognostic significance of CSF OCB in APCA—a marker of intrathecal inflammation—has not been systematically evaluated. While previous studies, such as those by Serrallach et al. (1) and Garone et al. (6), have provided valuable insights into the etiologies of pediatric ataxia, our study offers data from a large Chinese pediatric cohort, potentially reflecting regional differences in infectious triggers and vaccination practices. Furthermore, we specifically investigate the prognostic implications of CSF OCB in APCA and explore the spectrum of autoantibody-associated ataxias in our population, aspects that remain less defined.

We hypothesize that (1) the etiological distribution of acute ataxia varies significantly across age groups, with APCA predominating in younger children and immune-mediated or other complex disorders potentially increasing with age; and (2) CSF OCB positivity in APCA predicts a more protracted clinical course and higher relapse risk, reflecting underlying neuroinflammatory mechanisms.

To address these gaps, we retrospectively analyzed 257 children with acute ataxia from a single tertiary center, stratifying findings by age and etiology. This study aims to refine diagnostic algorithms and highlight high-risk features warranting further investigation and potentially earlier or more targeted intervention.

## Methods

### Study design and participants

This retrospective cohort study enrolled 257 children admitted to Hebei Children's Hospital between January 2018 and December 2024 who presented with acute ataxia as the initial symptom. Inclusion criteria comprised: (1) age ≤14 years; (2) symptom onset to hospital presentation within ≤2 weeks; and (3) ataxia as the primary presenting symptom. Ataxia was considered the “primary presenting symptom” if it was documented as the chief complaint or the most prominent and disabling sign in the admission notes, as determined by the initial evaluating physician's narrative. This operational definition serves to ensure that our cohort consists of children for whom ataxia was the central clinical problem driving their presentation, rather than a secondary feature of another dominant neurological syndrome. For added clarity, we specified that patients were excluded if their medical records indicated ataxia as a minor finding relative to other primary issues, such as severe encephalopathy or seizures at onset. Patients with incomplete core data precluding etiological determination were excluded. The study was approved by the Ethics Committee of Hebei Children's Hospital (Approval No. IRB-0159), with waiver of informed consent due to the retrospective nature of the analysis of de-identified data.

### Patient enrollment process

**Figure 1:**
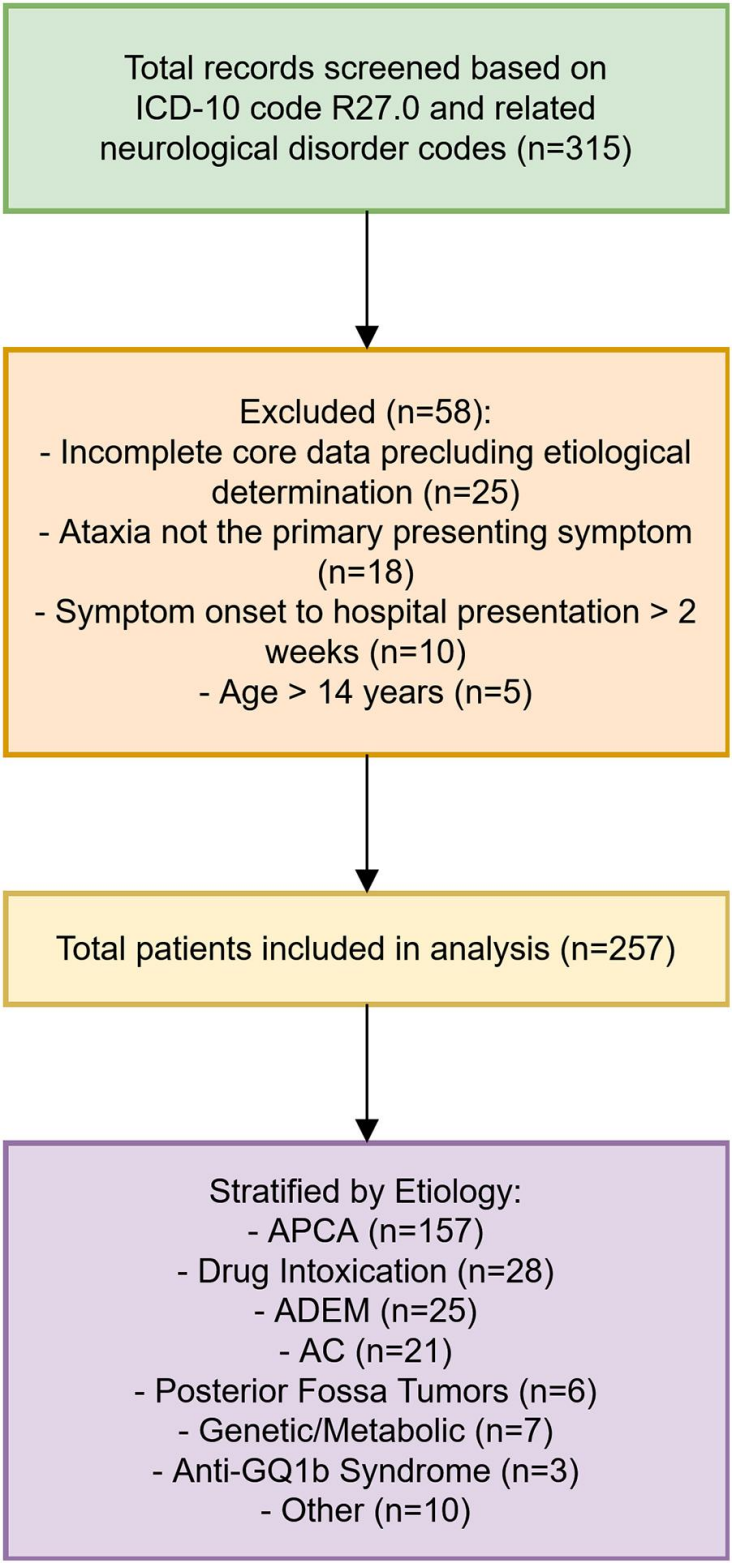
Flowchart of patient enrollment.

Potential cases were identified through the hospital's electronic medical record system using diagnostic codes for ataxia (ICD-10: R27.0) and by reviewing records of patients with related neurological disorder codes (e.g., encephalitis, encephalomyelitis) where ataxia was a prominent symptom documented in the clinical notes. Two neurologists independently reviewed medical records to verify eligibility based on predefined criteria. Discrepancies were resolved through consensus. Eligible patients were tracked from admission to discharge, with follow-up data collected via outpatient visits or telephone interviews for recurrent or unresolved cases. The patient selection process is illustrated in Figure 1.

### Data collection and variables

Clinical data extracted included demographic characteristics (age, sex), disease course (onset timing, prodromal infections), diagnostic investigations [cerebrospinal fluid (CSF) analysis, neuroimaging, serological/CSF autoantibodies, pathogen testing, toxicology screening], treatment regimens, hospitalization duration, outcomes, and follow-up records. Autoantibody screening (including MOG IgG, GQ1b, AQP4, and MBP antibodies, as well as CSF OCB) was not uniformly performed across all patients due to the retrospective nature of the study. It was generally conducted in patients with suspected immune-mediated etiologies, such as those presenting with features of ADEM, AC, recurrent ataxia, atypical clinical courses, or where initial investigations did not yield a clear diagnosis. A total of 135 patients underwent some form of autoantibody testing as detailed in the Results. Systemic tumor screening (e.g., abdominal ultrasound, urinary catecholamines) was performed in patients with refractory or relapsing ataxia, or those presenting with features suggestive of a paraneoplastic syndrome, such as opsoclonus-myoclonus.

### Diagnostic criteria

Diagnoses were established based on clinical features, laboratory findings, neuroimaging, and exclusion of other conditions, applying widely accepted criteria where available:

- **Acute Postinfectious Cerebellar Ataxia (APCA):** APCA was diagnosed based on acute onset of cerebellar ataxia (gait, truncal, and/or limb ataxia) within 4 weeks following a presumed viral or bacterial infection or vaccination, absence of encephalopathy or focal neurological signs beyond cerebellar dysfunction, normal or non-specific cranial MRI findings (or MRI not performed if clinically typical and other causes excluded), and exclusion of other identifiable causes (e.g., intoxication, structural lesions, metabolic disorders).
- **Acute Cerebellitis (AC):** AC was diagnosed by acute onset of cerebellar ataxia, often accompanied by fever, headache, vomiting, or altered consciousness, in conjunction with MRI evidence of cerebellar inflammation (e.g., swelling, T2/FLAIR hyperintensity, and/or contrast enhancement in the cerebellum) and/or CSF pleocytosis, after excluding other causes, including direct infectious cerebellitis confirmed by negative CSF pathogen testing (e.g., PCR for viral pathogens).
- **Acute Disseminated Encephalomyelitis (ADEM):** ADEM was diagnosed based on criteria adapted from the International Pediatric MS Study Group (IPMSSG) 2013 guidelines, requiring a polyfocal clinical CNS event with a presumed inflammatory demyelinating cause, typically including encephalopathy (alteration in consciousness or behavior unexplained by fever); no new clinical or MRI findings emerging 3 months or more after the initial event; and characteristic MRI findings [diffuse, poorly demarcated, large (>1–2 cm) lesions predominantly involving cerebral white matter, though deep grey matter, brainstem, and cerebellum could also be affected].
- **Drug Intoxication:** Diagnosis of drug intoxication was based on a clear history of exposure (accidental or intentional), positive toxicology screening where performed and available, and/or resolution of ataxia consistent with the drug's metabolism and withdrawal, in the absence of other identifiable causes for ataxia.
- **Other Etiologies:** Diagnoses such as posterior fossa tumors, genetic/metabolic disorders, and anti-GQ1b antibody syndrome were based on specific confirmatory investigations including neuroimaging, genetic/metabolic testing, and targeted antibody assays, respectively.

### Immunological assays

Cerebrospinal fluid (CSF) OCBs were detected by isoelectric focusing followed by immunoblotting against IgG. A Type II pattern (i.e., OCBs present in CSF but not in a paired serum sample) was considered indicative of intrathecal IgG synthesis and thus positive. Serum myelin oligodendrocyte glycoprotein immunoglobulin G (MOG-IgG) antibodies were tested using validated cell-based assays (CBAs) employing live cells transfected with full-length human MOG; a serum titer of ≥1:10 was considered positive. Anti-GQ1b antibodies were assessed by commercial enzyme-linked immunosorbent assay (ELISA) kits. Antibodies against myelin basic protein (MBP) and aquaporin-4 (AQP4) were tested using commercial ELISA kits, with positivity determined according to the manufacturers' instructions. In selected cases with suspicion of autoimmune encephalitis, a broader panel including anti-glutamic acid decarboxylase (GAD) and other relevant antibodies was performed based on clinical judgment.

### Treatment regimens

First-line immunotherapy for patients with severe symptoms or suspected inflammatory etiologies (e.g., severe APCA, AC, ADEM) typically consisted of intravenous methylprednisolone (IVMP) at a dose of 20–30 mg/kg/day (maximum 1 g/day) for 3–5 days, often followed by an oral prednisone taper over 2–4 weeks. Second-line treatments for refractory or relapsing cases included intravenous immunoglobulin (IVIG) or other immunomodulatory agents like rituximab, tailored to the individual patient's clinical course and diagnosis.

### Group stratification

Participants were categorized by age into: infants/toddlers (0–3 years), preschool (4–6 years), and school-age (7–14 years) groups for etiological distribution analysis. For clinical comparisons, cohorts were divided by diagnosis: acute postinfectious cerebellar ataxia (APCA) vs. acute cerebellitis (AC). Within the APCA group, patients were further classified by CSF oligoclonal band (OCB) status (Type II OCB as positive; others as negative) to assess prognostic differences.

### Bias mitigation

To minimize selection bias, consecutive enrollment of eligible inpatients was ensured during the study period. Measurement bias was reduced by standardized data extraction protocols and dual-physician adjudication of ambiguous cases. Confounding was addressed through stratified analyses by age and etiology.

### Statistical analysis

Data were analyzed using SPSS 27.0. Normality of continuous variables was assessed using the Shapiro–Wilk test. As data were found to be non-normally distributed, they were expressed as median (P25, P75) and compared via Mann–Whitney *U* test. Categorical variables were reported as counts (%) and analyzed with chi-square tests or Fisher's exact test where appropriate. Two-tailed *P* < 0.05 indicated statistical significance. Missing data for certain specialized investigations (e.g., CSF OCB, specific autoantibodies) were inherent to the retrospective design. Analyses involving these variables were conducted on the subset of patients with available data, as specified in the results and table footnotes. For primary outcome analyses where critical data were missing that precluded etiological determination, patients were excluded as per our exclusion criteria.

## Results

### Demographic characteristics

The study cohort comprised 257 pediatric patients presenting with acute ataxia as the primary symptom, including 137 males and 120 females (male-to-female ratio 1.14:1). The median age at onset was 3 years (range: 10 months to 14 years), with a mean age of 4.3 ± 3.38 years. Concurrent symptoms at presentation included fever in 35 cases (13.6%), headache or dizziness in 62 cases (24.1%), vomiting in 65 cases (25.3%), and encephalopathy in 54 cases (21%).

### Etiological distribution of acute ataxia

Among the 257 cases, the leading causes were acute postinfectious cerebellar ataxia (APCA, 157 cases, 61.1%), drug intoxication (28 cases, 10.9%), acute disseminated encephalomyelitis (ADEM, 25 cases, 9.7%), and acute cerebellitis (AC, 21 cases, 8.2%). Less frequent etiologies included posterior fossa tumors (6 cases, 2.3%), genetic/metabolic disorders (7 cases, 2.7%), anti-GQ1b antibody syndrome (3 cases, 1.2%), and other rare conditions (10 cases, 3.9%).

Among 157 APCA cases, 106 (67.5%) had preceding infections within 4 weeks, predominantly respiratory infections (102 cases). Pathogens were identified in 56 cases, including enteroviruses (14 cases), Mycoplasma pneumoniae (14 cases), respiratory syncytial virus (5 cases), influenza A virus (4 cases), varicella-zoster virus (3 cases), influenza B virus (3 cases), rhinovirus (2 cases), rotavirus (2 cases), coxsackievirus (2 cases), parainfluenza virus type 3 (1 case), Mycobacterium tuberculosis (1 case), norovirus (1 case), adenovirus (1 case), and SARS-CoV-2 (1 case). Six patients (3.8%) had recent vaccination history, including DTP vaccine (2 cases), EV71 vaccine (1 case), polio vaccine (1 case), varicella vaccine (1 case), and meningococcal vaccine (1 case). Among 21 AC cases, 10 (48%) had preceding infections within 4 weeks, with pathogens identified in 3 cases (Mycoplasma pneumoniae in 2 cases and respiratory syncytial virus in 1 case). Among 25 ADEM cases, 14 (56%) had preceding infections, with pathogens identified in 11 cases (Mycoplasma pneumoniae in 6 cases, EB virus in 3 cases, enterovirus in 1 case, and respiratory syncytial virus in 1 case).

### Drug intoxication and other etiologies

The 28 drug intoxication cases involved benzodiazepines (17 cases), carbamazepine (3 cases), promethazine (2 cases), flunarizine (2 cases), olanzapine (2 cases), difenidol (1 case), and amantadine (1 case). Among these, 23 cases were accidental ingestions, while 5 cases involved intentional drug misuse or suicide attempts. The 6 posterior fossa tumors included medulloblastoma (4 cases) and pilocytic astrocytoma (2 cases). The 7 genetic/metabolic disorder cases all had preceding respiratory infections within 1 week and included late-onset methylmalonic acidemia (3 cases), biotinidase deficiency (1 case), Leigh syndrome (1 case), hereditary cerebellar ataxia with ATP1A3 mutation (1 case), and adrenoleukodystrophy (1 case). Three cases presenting initially with acute ataxia and opsoclonus-myoclonus were subsequently diagnosed as paraneoplastic syndrome associated with adrenal neuroblastoma and were included in the “Other” category.

### Age-Stratified etiological patterns

The predominant causes varied significantly by age group. In infants and toddlers (0–3 years, *n* = 112), the leading causes were APCA (74 cases, 66.1%), drug intoxication (16 cases, 14.3%), and ADEM (9 cases, 8.0%). In preschool children (4–6 years, *n* = 89), the main causes were APCA (56 cases, 62.9%), ADEM (12 cases, 13.5%), and AC (7 cases, 7.9%). In school-age children (7–14 years, *n* = 56), the primary causes were APCA (27 cases, 48.2%), AC (12 cases, 21.4%), and drug intoxication (8 cases, 14.3%).

### Comparative analysis: APCA vs. Acute Cerebellitis

As detailed in Table 1, the 157 APCA cases demonstrated significant differences from 21 AC cases across multiple clinical parameters. AC patients had older onset age (median 9 vs. 3 years, *P* < 0.001), longer duration from symptom onset to hospital presentation (median 5 vs. 3 days, *P* = 0.023), longer hospital stays (median 17 vs. 11 days, *P* < 0.001), and higher frequencies of encephalopathy (81% vs. 18%, *P* < 0.001), headache (86% vs. 5.7%, *P* < 0.001), and vomiting (57% vs. 28.7%, *P* = 0.009). Cerebrospinal fluid analysis showed markedly elevated nucleated cell counts in AC (median 35 vs. 7 × 10^6^/L, *P* < 0.001). No statistically significant differences were observed in sex distribution (*P* = 0.521) or the proportion of patients with preceding infections (*P* = 0.072), and CSF oligoclonal band positivity rates were similar between groups in the tested subsets (*P* = 0.859).

**Table 1:** Comparative analysis of clinical characteristics between APCA and AC groups.

Characteristic	APCA group (*n* = 157)	AC group (*n* = 21)	Statistic	*P*-value
Onset Age (years), median (P25, P75)	3 (1.65, 4.75)	9 (5.5, 11)	*U* = 534.5	<0.001
Male [*n* (%)]	78 (49.7)	12 (57.1)	*χ*^2^ = 0.413	0.521
Onset Duration (days), median (P25, P75)*	3 (2, 9)	5 (2.5, 8.5)	*U* = 1,151	0.023
Hospital Stay (days), median (P25, P75)	11 (7, 14)	17 (15, 27)	*U* = 683	<0.001
Preceding Infection [*n* (%)]	106 (67.5)	10 (47.6)	*χ*^2^ = 3.23	0.072
Symptoms [*n* (%)]				
Encephalopathy	28 (17.8)	17 (81.0)	*χ*^2^ = 39.064	<0.001
Headache	9 (5.7)	18 (85.7)	*χ*^2^ = 92.083	<0.001
Vomiting	45 (28.7)	12 (57.1)	*χ*^2^ = 6.902	0.009
CSF Nucleated Cells (×10^6^/L)**, median (P25, P75)	7 (2, 16)	35 (5, 142.25)	*U* = 763	<0.001
CSF OCB Positive [n (%)]***	24/77 (31.2)	6/18 (33.3)	*χ*^2^ = 0.032	0.859

APCA, acute postinfectious cerebellar ataxia; AC, acute cerebellitis; OCB, oligoclonal bands; CSF, cerebrospinal fluid. Data presented as -median (P25, P75) or *n* (%). *U* = Mann–Whitney *U* test value; *χ*^2^ = Chi-square test value.

* “Onset Duration” refers to the duration from symptom onset to hospital presentation.

** APCA group *n* = 147; AC group *n* = 20 for CSF Nucleated Cells.

*** APCA group *n* = 77; AC group *n* = 18 for CSF OCB testing.

### Immunological profiling in APCA subgroups

Among 77 APCA patients tested for oligoclonal bands (OCB), 24 (31.2%) were positive. Compared to OCB-negative patients (*n* = 53), the OCB-positive subgroup (*n* = 24) exhibited older onset age (median 4 vs. 3 years, *P* = 0.037), prolonged hospitalization (median 15 vs. 12 days, *P* = 0.003), higher CSF nucleated cell counts (median 12.5 vs. 4 × 10^6^/L, *P* = 0.007), and a significantly increased relapse rate (5/24, 20.8% vs. 0/53, 0%, *P* = 0.001) as shown in Table 2. Steroid utilization rates were comparable between groups (87.5% vs. 71.7%, *P* = 0.129).

**Table 2:** Comparative analysis of clinical characteristics between OCB-positive and OCB-negative APCA patients.

Characteristic	OCB-Negative APCA (*n* = 53)	OCB-Positive APCA (*n* = 24)	Statistic	*P*-value
Onset Age [years], median (P25, P75)	3 (2, 4)	4 (3, 7)	*U* = 448.5	0.037
Male [n (%)]	29 (54.7)	12 (50.0)	*χ*^2^ = 0.148	0.701
Onset Duration [days], median (P25, P75)*	2 (0.725, 4)	3 (2, 5)	*U* = 423.5	0.018
Hospital Stay [days], median (P25, P75)	12 (8, 15)	15 (12, 20)	*U* = 369	0.003
Preceding Infection [n (%)]	38 (71.7)	18 (75.0)	*χ*^2^ = 0.091	0.763
CSF Nucleated Cells (×10^6^/L), median (P25, P75)	4 (2, 14)	12.5 (6.25, 34.25)	*U* = 392	0.007
Steroid Use [*n* (%)]	38 (71.7)	21 (87.5)	*χ*^2^ = 2.303	0.129
Relapse [*n* (%)]	0 (0)	5 (20.8)	*χ*^2^ = 11.808 (Fisher’s exact test)	0.001

OCB, oligoclonal bands; APCA, acute postinfectious cerebellar ataxia; CSF, cerebrospinal fluid. Data presented as median (P25, P75) or *n* (%). *U* = Mann–Whitney *U* test value; *χ*^2^ = Chi-square test value.

* “Onset Duration” refers to the duration from symptom onset to hospital presentation.

### Autoantibody profiles

Comprehensive antibody testing was performed in a subset of 135 patients based on clinical suspicion. Among APCA patients, CSF OCB positivity was found in 24/77 tested cases, and elevated serum myelin basic protein (MBP) antibodies were detected in 7/77 tested cases. In the AC subgroup, 6/18 tested cases showed CSF OCB positivity, with no other specific autoantibodies identified in this group from the panel tested. Among 25 ADEM cases (with varying numbers tested for specific antibodies), 11/25 were serum MOG-IgG positive, 8/25 had elevated MBP antibodies, 8/25 showed CSF OCB positivity, and 1/25 (where tested) was positive for AQP4-antibody. Three patients presenting with isolated ataxia demonstrated CSF GQ1b-antibody positivity (diagnosed with anti-GQ1b antibody syndrome). Additionally, three MOG-IgG positive cases presented with acute cerebellar ataxia and preceding respiratory infections, showing mild CSF pleocytosis and normal initial cranial MRI; two of these three cases experienced recurrence during follow-up. Screening for specific autoimmune cerebellar ataxias, including tests for anti-GAD antibodies, was negative in all tested patients.

### Neuroimaging findings

**Figure 2:**
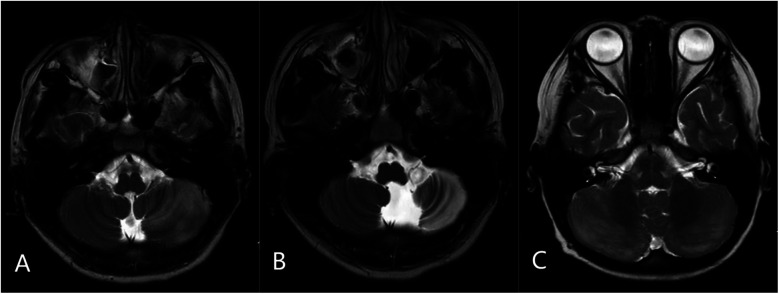
Representative MRI findings in acute cerebellitis (AC): **(A)** axial T2-weighted image showing diffuse hyperintensity and swelling in the left cerebellar hemisphere. **(B)** Follow-up axial T2-weighted image at 2 years in the same patient demonstrating left cerebellar atrophy. **(C)** Axial T2-weighted image from another patient showing bilateral cerebellar diffuse hyperintensity with swelling.

Neuroimaging analysis of 210 cases showed no characteristic abnormalities in most APCA patients, with MRIs being either normal or showing non-specific findings. AC cases (*n* = 21) exhibited cerebellar T2 hyperintensity with swelling (bilateral in 11/21, unilateral in 10/21), including three cases with tonsillar involvement (one showing herniation) and four cases with concurrent supratentorial basal ganglia lesions. Among eight AC cases with follow-up MRI at 1–2 years, three developed cerebellar atrophy; these three patients experienced persistent neurological sequelae, including moderate ataxia in two and significant dysarthria in one. The five cases showing complete radiological resolution had corresponding good clinical outcomes with minimal or no residual symptoms at last follow-up (Figure 2).

### Clinical follow-up and outcomes

Five OCB-positive APCA patients experienced disease relapse following initial steroid response. Immunotherapy (IVIG and/or subsequent rituximab in one case) achieved remission in four cases, while one OCB-positive patient who experienced multiple relapses was subsequently diagnosed with neuroblastoma upon the third relapse. The remaining four OCB-positive relapsing cases showed no evidence of neoplasia during their follow-up period (median 24 months, range 12–36 months).

## Discussion

Dysfunction in neural pathways involving the cerebellum, basal ganglia, cerebral cortex, spinal cord, sensory peripheral nerves, or vestibular system can lead to ataxia, with cerebellar dysfunction being the primary cause in children (7). In this study, acute postinfectious cerebellar ataxia (APCA), acute cerebellitis (AC), drug intoxication, and acute disseminated encephalomyelitis (ADEM) constituted the major etiologies of acute childhood ataxia, accounting for nearly 90% of cases. APCA was more prevalent in children aged 0–6 years, while AC was more common in school-aged children. Both APCA and AC typically occurred within a few days to weeks [median 10–15 days in some reports (8)] after infection or vaccination. The most frequently identified pathogens in our cohort were enteroviruses, Mycoplasma pneumoniae, respiratory syncytial virus, and influenza viruses (types A and B), collectively accounting for a significant proportion of cases where a pathogen was identified. Other pathogens included varicella-zoster virus, rhinovirus, rotavirus, coxsackievirus, parainfluenza virus type 3, norovirus, and adenovirus. This distribution differs from some international reports, where varicella-zoster virus and mumps virus predominate (1), likely reflecting differences in vaccination coverage. In China, the implementation of a two-dose varicella vaccination schedule (first dose at 18 months, second dose at 4 years) has achieved >80% coverage for the first dose and 40%–73% for the second dose in urban areas like Beijing, with vaccine effectiveness reaching 94% (9). Concurrently, mumps-related cases have declined nationally following the inclusion of measles-mumps-rubella (MMR) vaccine in the Expanded Program on Immunization in 2008 (10). These vaccination policies likely explain the reduced prevalence of varicella-zoster and mumps-associated ataxia in our cohort, while enteroviruses—for which no widely available vaccine covering all serotypes exists—remain prominent.

Drug intoxication represented the second most common cause of acute ataxia, with benzodiazepines, anticonvulsants (e.g., carbamazepine), and antipsychotics (e.g., olanzapine) being the most frequently implicated agents, consistent with other studies (6). Age distribution showed a bimodal pattern, with accidental ingestions predominating in children under 3 years and intentional misuse/suicide attempts peaking in adolescents. Notably, unexplained acute ataxia in adolescents should raise suspicion for drug intoxication, particularly in those with underlying anxiety or depressive disorders.

Of particular interest, seven cases (2.7%) of genetic/metabolic disorders presented with acute ataxia, all triggered by concurrent infections. Certain inborn errors of metabolism may manifest as episodic or intermittent ataxia during metabolic stressors such as infection, fasting, or physiological stress. These episodes result from impaired energy metabolism, accumulation of toxic metabolites, or substrate deficiencies. A history of developmental delay or seizures may provide critical diagnostic clues for underlying metabolic disorders in children with acute ataxia (11), as seizures and developmental regression are key clinical indicators that should prompt consideration of inherited metabolic disorders (12, 13). Notably, developmental delay occurs in approximately 60% of patients with metabolic epilepsy (12), reinforcing its diagnostic significance.

While intracranial tumors have been reported as common causes of childhood ataxia in previous studies, particularly given that posterior fossa tumors (PFTs) account for approximately 50% of all childhood brain tumors and frequently manifest with ataxia as a predominant symptom at presentation (14, 15), only six cases (2.3%) of posterior fossa tumors were identified in our cohort. This lower-than-expected prevalence likely reflects selection bias, as our study exclusively enrolled neurology inpatients. Some tumor cases may have been identified and managed by neurosurgery or oncology departments directly. Furthermore, our identification of three cases of paraneoplastic opsoclonus-myoclonus-ataxia syndrome underscores the necessity of considering underlying malignancy in children with atypical or refractory ataxia, even when ataxia is the most prominent feature at onset. Additionally, conditions such as muscle weakness, hypotonia, or emotional disorders may mimic ataxia (“pseudoataxia”), necessitating careful clinical differentiation.

International retrospective studies highlight acute cerebellitis as a potentially life-threatening condition, with clinical courses ranging from benign presentations to severe complications such as intracranial hypertension, cerebellar tonsillar herniation, and obstructive hydrocephalus. Approximately one-third of patients may develop neurological sequelae, including persistent ataxia, dysmetria, movement disorders, memory impairment, dysarthria, or tremor (1, 16). MRI remains the diagnostic modality of choice, typically revealing patterns of bilateral or unilateral hemispheric cerebellitis, sometimes with concurrent encephalitis. Follow-up imaging may demonstrate cerebellar volume loss (17). Our comparative analysis between AC and APCA groups suggests that children aged >5 years with a longer duration of symptoms before presentation, encephalopathy, headache, vomiting, or abnormal CSF mononuclear cell counts should undergo prompt MRI and consideration for early therapeutic intervention. Notably, only one case in our AC cohort developed tonsillar herniation, and none required surgical intervention or developed severe, disabling long-term sequelae, possibly reflecting the benefits of aggressive immunotherapy.

Oligoclonal bands (OCBs), representing antibodies against diverse antigens, are well-established immunopathological markers in multiple sclerosis and other neuroinflammatory disorders (18). According to international criteria, type II OCBs (CSF-restricted IgG) indicate intrathecal antibody synthesis, reflecting compartmentalized humoral immune responses. In our cohort, approximately one-third of tested APCA and AC cases demonstrated OCB positivity. Comparative analysis revealed that OCB-positive APCA patients were older (>3 years), had prolonged hospitalization, and exhibited higher CSF nucleated cell counts—all features consistent with a more robust inflammatory pathophysiology. Strikingly, OCB-positive cases showed significantly higher relapse rates, although this observation is based on a small number of relapsing patients (*n* = 5) and requires validation in larger, prospective studies. One potential explanation for this tendency is persistent immune activation (19). OCBs mark sustained B-cell clonal expansion within the CNS (20), a process also linked to higher relapse rates in conditions like pediatric multiple sclerosis (21). This may stem from incomplete immunomodulation or dysregulated immune feedback mechanisms (22). While most relapses responded completely to immunotherapy, one patient required rituximab after failing first-line treatments. This case did not fully meet diagnostic criteria for either APCA or primary autoimmune cerebellar ataxia (23), underscoring the need for refined diagnostic frameworks. Another OCB-positive patient was subsequently diagnosed with neuroblastoma upon third relapse. However, a prior study of opsoclonus-myoclonus syndrome (OMS) found no association between OCB positivity and neuroblastoma (24), suggesting that the relationship between OCBs and paraneoplastic ataxias requires further investigation.

Beyond OCBs, myelin oligodendrocyte glycoprotein (MOG) IgG antibodies represented the most frequently detected autoantibodies in our cohort. MOG antibody-associated diseases demonstrate remarkable phenotypic diversity, with ADEM being the most common presentation in children (25). Other manifestations include optic neuritis, myelitis, and brainstem/cerebellar involvement (25). We identified 14 MOG IgG antibody-positive cases, including 11 with ADEM and three with isolated cerebellar ataxia (two with relapsing courses). Although MOG antibody-associated pure cerebellar ataxia with normal imaging is exceptionally rare, it may constitute a distinct phenotype meriting inclusion in the differential diagnosis of childhood acute ataxia (26), particularly in recurrent cases. Myelin basic protein (MBP), an immunogenic component of central myelin, triggers antibody responses upon release during demyelination (27). In our study, MBP antibodies frequently coexisted with MOG antibodies and OCBs, collectively implicating immune-mediated mechanisms in these cases.

Anti-GQ1b antibody syndrome, initially described in Miller-Fisher syndrome (MFS), is now recognized in variants exhibiting partial MFS features (28). Acute sensory ataxic neuropathy represents one such variant, characterized by cerebellar-like ataxia without ophthalmoplegia, resulting from antibody binding to sensory neurons in spinocerebellar tracts (29). While electrodiagnostic studies may aid diagnosis, none of our three cases showed characteristic electrophysiological abnormalities, possibly reflecting suboptimal testing timing. The prognosis is typically favorable, with low relapse rates (30).

This study has several limitations inherent to its single-center, retrospective design. As enrollment was restricted to neurology inpatients, cases managed exclusively in outpatient or emergency settings (e.g., mild intoxications, trauma, stroke, or surgically treated tumors presenting directly to other departments) were likely excluded, potentially introducing selection bias and an underestimation of these etiologies. This inpatient focus might also select for more severe or complex cases. Second, despite using standardized criteria, the retrospective nature could lead to diagnostic misclassification in some ambiguous cases. Third, comprehensive long-term outcome data with standardized scales were not available for all patients, limiting our ability to draw definitive conclusions about prognosis or fully assess the dynamic changes of ataxia over time. Fourth, specialized investigations like autoantibody testing were not uniformly performed but based on clinical suspicion, which may affect the reported frequencies of these conditions. Finally, our primary reliance on the ICD-10 code for ataxia (R27.0) for initial screening, despite supplementary searches, might have led to missing some patients whose ataxia was documented but coded under a different primary diagnosis.

Our findings underscore that childhood acute ataxia warrants tailored diagnostic approaches based on age, history, and examination findings to facilitate early diagnosis and optimize resource utilization. For cases suggesting central demyelination or recurrent ataxia, comprehensive evaluation including autoantibody testing, neoplastic screening, and metabolic/genetic investigations is essential. The observed etiological spectrum and clinical trajectories align with international reports while highlighting region-specific variations in pathogen distributions, likely reflecting differential vaccination practices.

## Conclusion

This study delineates APCA, AC, drug intoxication, and ADEM as the predominant causes of acute childhood ataxia, with age-specific etiological patterns. CSF OCB positivity may identify an APCA subgroup with more pronounced inflammatory features and a potentially higher relapse risk, a finding that warrants further investigation. MOG IgG antibodies represent prevalent autoantibodies beyond OCBs, including rare presentations of isolated cerebellar ataxia. These findings emphasize the importance of judicious diagnostic stratification and immunological profiling in pediatric ataxia.

## Data Availability

The original contributions presented in the study are included in the article/Supplementary Material, further inquiries can be directed to the corresponding author.
